# Insights from 24-hour actigraphy using functional linear modeling in children with and without ADHD

**DOI:** 10.1038/s41598-025-24040-5

**Published:** 2025-10-23

**Authors:** Mirjam Ziegler, Pascal Maurice Aggensteiner, Katja Becker, Manfred Döpfner, Julia Geissler, Sarah Hohmann, Reto Huber, Christine Igel, Karina Janson, Konstantin Mechler, Sabina Millenet, Marcel Romanos, Daniel Brandeis, Tobias Banaschewski, Anna Kaiser

**Affiliations:** 1 Department of Child and Adolescent Psychiatry and Psychotherapy, Central Institute of Mental Health, Medical Faculty Mannheim, Heidelberg University, Mannheim, Germany; 2 German Center for Mental Health (DZPG), partner site Mannheim-Heidelberg-Ulm, Mannheim, Germany; 3 Department of Child and Adolescent Psychiatry, Psychosomatics and Psychotherapy, University of Marburg and University Hospital Marburg (UKGM), Marburg, Germany; 4 Center for Child and Adolescent Cognitive Behavior Therapy (CEKiP), Faculty of Medicine and University Hospital Cologne, University of Cologne, Cologne, Germany; 5 Department of Child and Adolescent Psychiatry, Psychosomatics and Psychotherapy, Faculty of Medicine and University Hospital Cologne, University of Cologne, Cologne, Germany; 6 Department of Child and Adolescent Psychiatry, Psychosomatics and Psychotherapy, University Hospital Wuerzburg, Wuerzburg, Germany; 7 Department of Child and Adolescent Psychiatry, Psychotherapy and Psychosomatics, University Medical Centre Hamburg-Eppendorf, Hamburg, Germany; 8 Child Development Center and Pediatric Sleep Disorders Center, University Children’s Hospital Zürich – Eleonore Foundation, University of Zürich, Zurich, Switzerland; 9 Department of Child and Adolescent Psychiatry and Psychotherapy, Psychiatric Hospital, University of Zürich, Zurich, Switzerland; 10 Institute of Medical Psychology and Medical Sociology, Schleswig-Holstein, University Medical Center, Kiel University, Kiel, Germany; 11 Neuroscience Center Zurich, University and ETH Zurich, Zurich, Switzerland; 12 Center for Mind, Brain and Behavior (CMBB), University of Marburg and Justus Liebig University Giessen, Marburg, Germany

**Keywords:** 24h-actigraphy, Attention-deficit/hyperactivity disorder (ADHD), Functional linear modeling (FLM), Circadian rhythmicity, Children, ESCAlife, Psychology, ADHD

## Abstract

Sleep problems and alterations in circadian rhythmicity have been widely reported in attention-deficit/hyperactivity disorder (ADHD) patients, but studies covering the full 24h-motor-activity patterns to explore whole-day activity cycles are scarce. Furthermore, correlates and predictors of (hyper-)activity patterns are largely unknown. A subsample of 74 children (6–12 years) with ADHD (*n* = 35) participating in the multicenter study ESCAlife and *n* = 39 controls recruited in one center were assessed using 24h-actigraphy for two weeks. Functional linear modeling (FLM) was applied to the actigraphy data and further post-hoc correlational analyses were conducted. FLM revealed no significant differences in 24h-actigraphy profiles between children with and without ADHD, but differentiated between ADHD presentations (combined vs. inattentive) around sleep onset time (around 8:00p.m.) on free days. In the ADHD group, the 24h-actigraphy profiles were associated with measures of the chronotype and early regulation problems in toddler age; in the control group, with daylight exposure. Finding 24h-activity profile differences between ADHD presentations, but not between ADHD and control children as in classical analyses of sleep-onset latency suggests that comprehensive 24h-FLM analyses and focused sleep-related analyses of actigraphy data capture complementary aspects altered in ADHD and its presentations. Another limitation is that the majority of children with ADHD were medicated. However, actigraphy may still prove valuable as a supplementary diagnostic tool in clinical ADHD assessment.

**Supplementary Information:**

The online version contains supplementary material available at 10.1038/s41598-025-24040-5.

## Introduction

ADHD is a neurodevelopmental disorder characterized by core symptoms in the domains inattention and/or hyperactivity/impulsivity^1^. In clinical routine and according to current clinical guidelines^2^, the diagnosis should be based on information derived from a clinical interview and questionnaires assessing symptom severity and impairment in multiple settings as reported by e.g. the affected individuals themselves (depending on age), parents/important caregivers, or teachers. To date, due to a lack of evidence of sufficient sensitivity and specificity, there are no objective markers that are recommended to be used in the standard diagnostic process.

However, objective measures might help to better characterize individual impairments in the sense of additional diagnostic information (leading to incremental validity) and, consequently to identify relevant treatment targets within a personalized medicine framework. For example, actigraphs might be used to objectively assess symptoms of overt motor (hyper-)activity at high resolution over long periods of time. Those devices offer the possibility to track symptoms of hyperactivity in the patient’s natural environment as well as during regular daily life activities. Porrino’s early work was among the first to demonstrate significant differences in motor activity between hyperactive children and healthy controls regardless of the time of day^3^. Subsequent studies employing actigraphy in children with ADHD primarily focused on sleep characteristics and circadian rhythmicity. Results showed alterations in ADHD when compared to typically developing peers^4–10^. These studies found that a significantly higher proportion of children and adults with ADHD suffers from sleep problems^4,7,9–14^ such as falling asleep late, waking up often during the night or having problems with getting up early. In our own recently published actigraphy study, which focused on sleep parameters in a subgroup of the current study^10^, children with ADHD aged 6–11 years showed longer sleep onset latency (SOL), higher intra-individual variability (IIV) in SOL, more movements during sleep, lower sleep efficiency, and a slightly larger sleep deficits on school days compared to free days. These findings are in line with previous studies that found sleep-onset problems and greater IIV in SOL in ADHD children compared to typically developing peers (e.g^15^, shorter total sleep time and lower sleep efficiency^5,7^. However, previous studies have predominantly focused on nighttime sleep patterns, neglecting the 24h-sleep-wake cycle and overall daily motor activity patterns. These patterns can potentially provide additional relevant information for optimizing the diagnostic process and treatment planning. To explore the 24h sleep-wake cycle, the actigraph needs to be worn continuously, both day and night, on school days and free days (i.e., weekends and holidays). This approach captures real-time movements throughout the entire 24h cycle, allowing for the measurement of physical activity during wakefulness and sleep in an ecological and naturalistic way^16^.

In one previous study, Tonetti et al.^17^ examined the 24h-actigraphy profile of adult ADHD patients using functional linear modeling (FLM^18^, FLM extends standard linear regression to the analysis of Fourier base functions modeling and smoothing the raw activity values^19^. They found significantly higher motor activity in patients than in controls from 4:00 to 7:00 a.m. and from 12:00 to 6:00 p.m. They concluded that the absence of the post-lunch dip during afternoon as well as the higher activity during the night in ADHD patients may be the result of lower homeostatic sleep pressure (for more details regarding homeostatic and circadian sleep processes see e.g^20^. In line with the results found for the adult ADHD group^17^, a pilot study^16^ comparing non-medicated ADHD children to typically developing peers revealed higher motor activity in the ADHD group. This increased activity was particularly seen during a window in the nighttime (2:00–3:00 a.m.) in addition to a general higher mean whole day activity for the patient group. Further, this pilot study found differences in the 24h-actigraphy profiles for the distinct ADHD presentations: children with predominantly hyperactive/impulsive and combined presentations showed significantly higher activity in the evening (around 06:00 p.m. to 09:00 p.m.) and around 03:00 a.m. than children with a predominantly inattentive presentation. Thus, the increased activity in the evening could correspond with the previously reported problems, such as deficits in falling asleep (SOL alterations) in children with ADHD, particularly those with the hyperactive/impulsive presentation. However, due to the small sample size of the subgroups, the results need to be interpreted with caution.

So far, specific factors related to and influencing the continuous 24h-actigraphy profile are not yet found. Identifying those factors might reveal important treatment targets, providing highly relevant and objective information, and might prove helpful with regards to treatment planning and personalization as well as (early) prevention of sleep problems.

One concept that might be linked to the 24h-activity profile is the chronotype as an important behavioral and psychological manifestation of variations in circadian functioning. The late chronotype (‘night owls’) falls asleep late and gets up late in contrast to early chronotypes or ‘early birds’, who tend to go to bed early and wake up early. The chronotype changes during lifetime and across developmental stages, with an early chronotype in childhood peaking latest in adolescence, stabilizing during adulthood and getting early again in elderly persons^21^. To assess the chronotype questionnaires^22^ like the Munich Chronotype Questionnaire (MCTQ^23^), have mainly been used, which has proven to be a valid measure^24^. Overall, patients with ADHD show a tendency towards the so called ‘late chronotype’^25–27^.

The circadian system in general mainly depends on external zeitgebers to keep the circadian rhythm aligned to the 24h-light-dark rhythm. Thereby, light represents the most important environmental zeitgeber^6,28^. Light also influences melatonin production^4,29^, which is associated with the total sleep time of children with ADHD^30^. Consequently, chronotype as well as external factors such as light exposure might relate to 24h-motor activity, but studies exploring those associations are lacking.

With regard to possibilities for early interventions and prevention from sleep problems, it might be interesting to identify early indicators of altered 24h-activity profiles. In the literature focusing on sleep problems, several precursor symptoms have already been discussed. Above all, early regulatory problems like excessive crying, sleep issues or feeding problems in early childhood have been reported as probably relevant early indicators related to later ADHD symptoms (e.g. hyperactivity) and sleep problems^10,31–33^. Additionally, actigraphy studies in preschoolers highlighted the role of efficient and sufficient sleep as possible indicator of (later developing) ADHD^34,35^ or the development of executive functions in general^36^. In contrast other studies failed to find a significant difference in actigraphy sleep measures between preschoolers with ADHD symptoms and controls^37^. Nevertheless, studies are scarce, especially concerning the association of early regulatory difficulties with 24h-motor activity patterns.

Given a lack of studies exploring 24h-actigraphy profiles in children with ADHD, analyzing the 24h whole-day motor activity pattern might provide relevant information that could be used in the diagnostic process and with regards to the implementation of more targeted interventions. In addition, exploring factors associated with 24h-actigraphy profiles could help to better understand the link between different theoretical constructs as well as the complexity of ADHD symptoms, subgroups and the heterogeneity within this diagnostic category. Respective findings might provide insights in day- and nighttime fluctuations which might help to characterize critical daily periods and relevant timepoints for targeted (early preventive) interventions.

Based on the previously reported findings, we hypothesized to find higher activity during the afternoon and in the nighttime in children with ADHD compared to their typically developing peers. Additionally, we expected to find higher activity in the combined ADHD presentation compared to the inattentive presentation. While several correlates and predictors of motor activity especially during the night/sleep have already been discussed in the literature (see e.g^4,10,28,31–33^) , we expect to find higher activity in the evenings in late chronotypes and in the mornings in early chronotpyes as well as more daily light exposure being related to less activity during the night. Moreover, we expected that current (see e.g^10^) and early sleep problems (as a potential precursor, see^31–33^) in toddler age are related to more motor activity during the night.

## Methods

### Participants

A total of *N* = 74 children aged 6–12 years participated in the study. Of these, *n* = 35 children met the diagnostic criteria for ADHD and were compared to *n* = 39 typically developing peers (TD; see Tables 1, 2 for details). Patients were recruited within the ESCAlife (Evidence-based, Stepped Care of ADHD along the life span) consortium, more precisely via the ESCAschool multicenter study investigating stepped-care treatments for school-aged children with ADHD (for more detail, see^38^). At the Mannheim site, actigraphy and 24h-rhythm data were recorded for 14 consecutive days in a subgroup of patients (partially already medicated children who subsequently received intensive treatment consisting of psychotherapy or neurofeedback). Furthermore, TD children were also recruited at the Mannheim site only. Written informed consent was obtained from the participating children and their parents/legal representatives and ethical approval was granted by the local ethics committee. For the ADHD patients, a formal diagnosis of ADHD had to be confirmed. Details on exclusion criteria have been published previously (e.g^10,38^) and are listed in Supplement 1.

### ADHD diagnosis and comorbidities

ADHD symptoms were assessed in a medication-free status based on a parent interview using the clinician-rated ADHD Checklist (Diagnostic System for Mental Disorders in Children and Adolescents (DISYPS-III^39^), Diagnose-Checklist ADHS (DCL–ADHS–clinical interview)) prior to any study-associated treatment or measurement. For more details, see Supplement 1. Furthermore, ADHD symptoms ratings from the parents and — if consent was obtained — from teachers were included using clinical questionnaires (FBB–ADHS–Parent + Teacher). All children were screened for psychiatric comorbidities (DCL-SCREEN-clinical interview, including DCL-DES, DCL-ASKS, DCL-ANG, DCL-ZWA, DCL-TIC).

### 24h-activity profile

The 24h-activity profile was recorded using actigraphs (ActiGraph LLC, Pensacola, FL, USA, model: wGT3X-BT) worn on the non-dominant wrist for 14 consecutive days and nights (24h) to ensure that enough free days and school days^40^ were included. Children were instructed to take off the actigraph only for showering, bathing, and sports. The ActiLife software (version 6.13.4) was used to extract minute-by-minute raw motor activity counts across 24h.

Additionally, daily self-report diaries (paper-pencil) were completed by children and parents to assess daily activities and to validate bedtimes and times the actigraphs were not worn.

Days with more than 120 min of non-wearing time (based on consecutive zeros in data sets) were excluded (e.g^41)^. For the analysis, all three axes (vertical, medio-lateral and anterior-posterior) were used (indicating vector magnitude) to detect activity. Data were analyzed separately for school days and free days.

### Chronotype and light exposure

We used the Munich ChronoType Questionnaire for children (MCTQ^23)^, MSFsc index—midsleep on free days, corrected for oversleeping) to assess the self-reported chronotype and light exposure during school days and free days.

### Sleep at toddler age and current sleep problems

To assess earlier sleep problems at toddler age (18–36 months), one retrospective question (‘the child had difficulties falling and staying asleep’) from a short version of the Revised Dimensions of Temperament Survey (DOTS-R^42^); questionnaire was used. To additionally assess current sleep problems, one item (‘difficulties with sleeping’) of the Child Behavior Checklist (CBCL/6-18R) was used.

### Statistical analyses

**Fig. 1:**
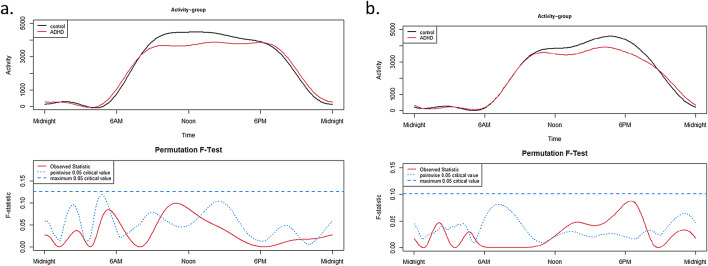
24h-activity profiles of ADHD and TD group on schooldays (**a**) and on free days (**b**).

**Fig. 2:**
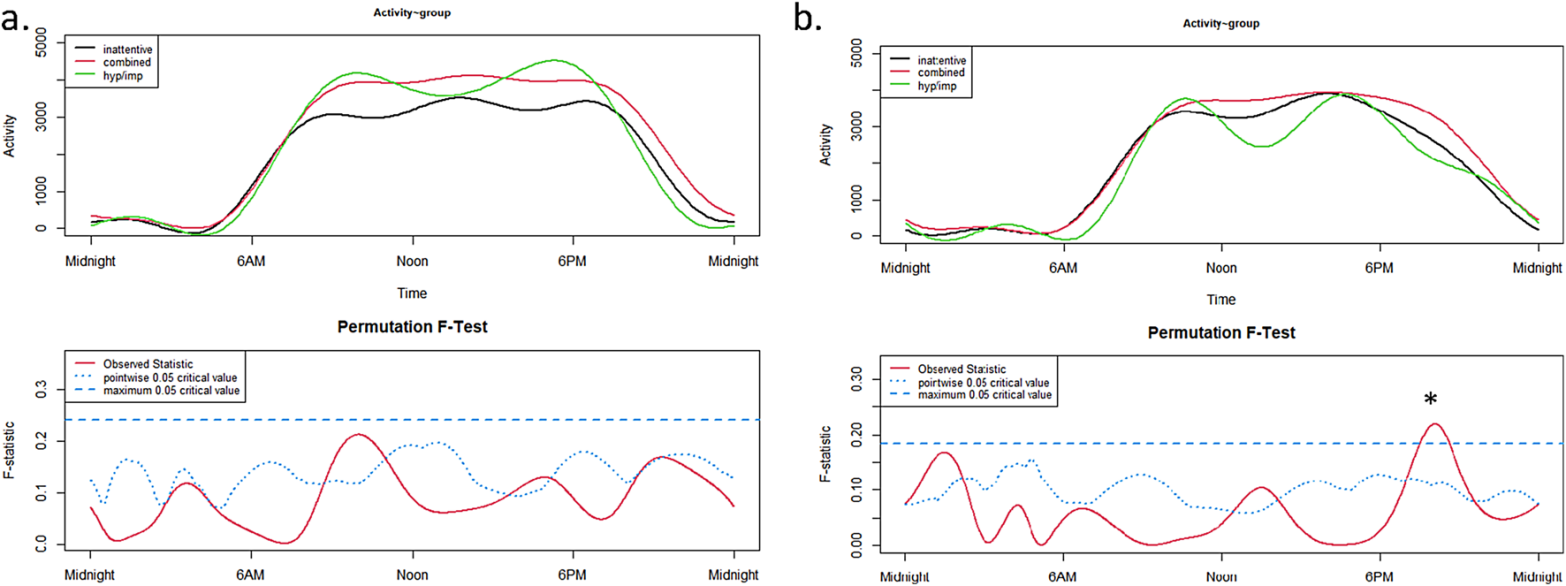
24 h-activity profiles of ADHD presentations on schooldays (**a**) and on free days (**b**).

**Fig. 3:**
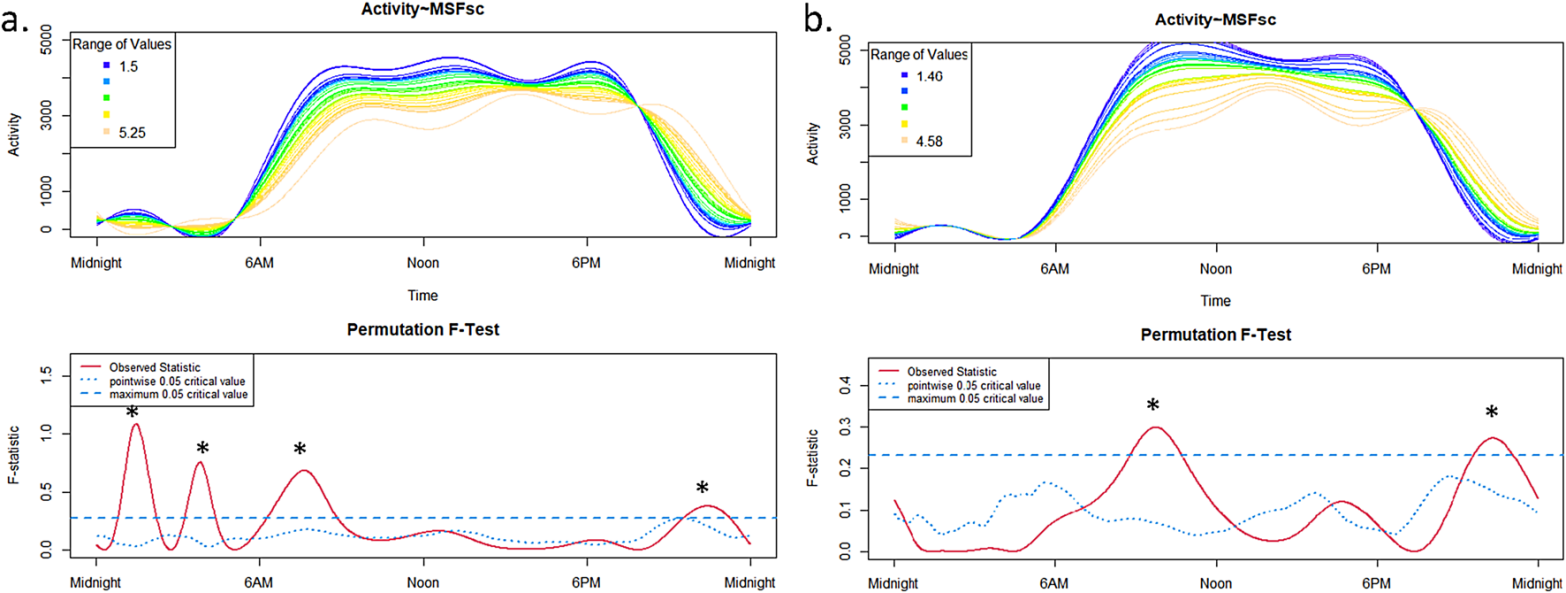
Schooldays: Influence of the chronotype (MSFsc) in the ADHD group (**a**) and in the TD group (**b**) on 24 h-activity profiles.

**Fig. 4:**
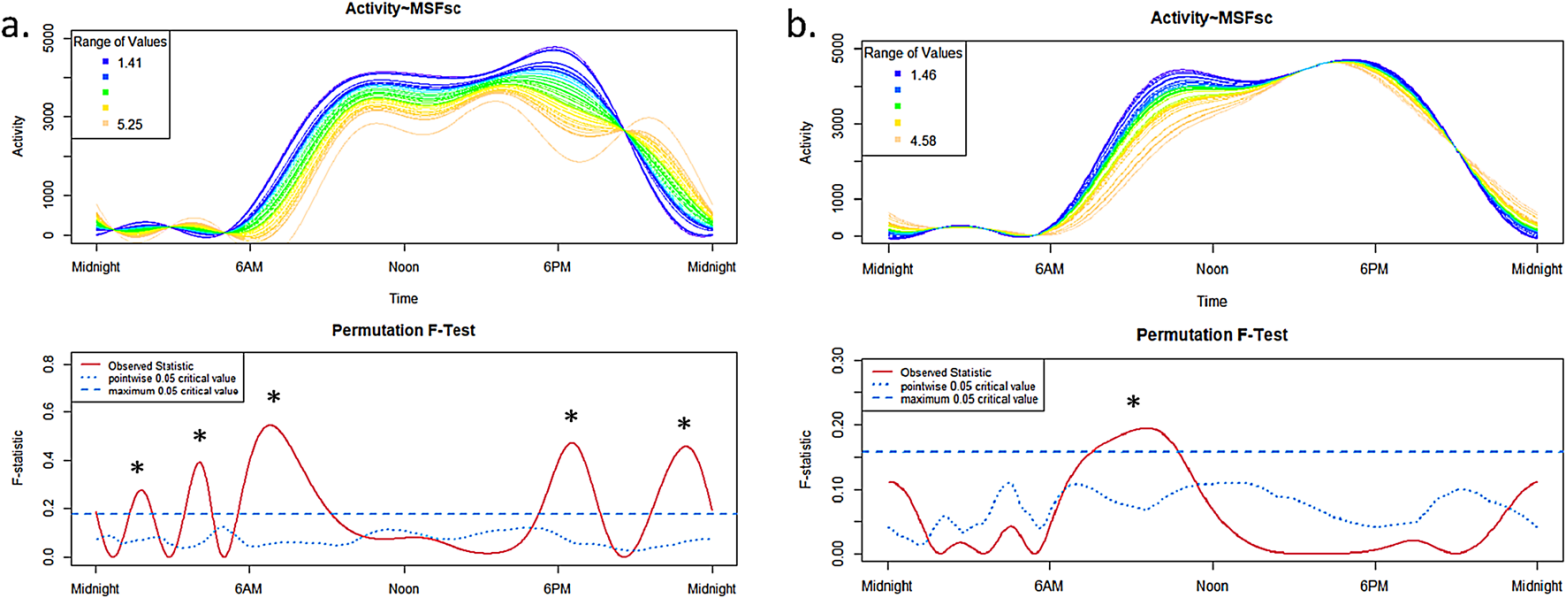
Free days: Influence of the chronotype (MSFsc) in the ADHD group (**a**) and in the TD group (**b**) on 24h-activity profiles.

**Fig. 5:**
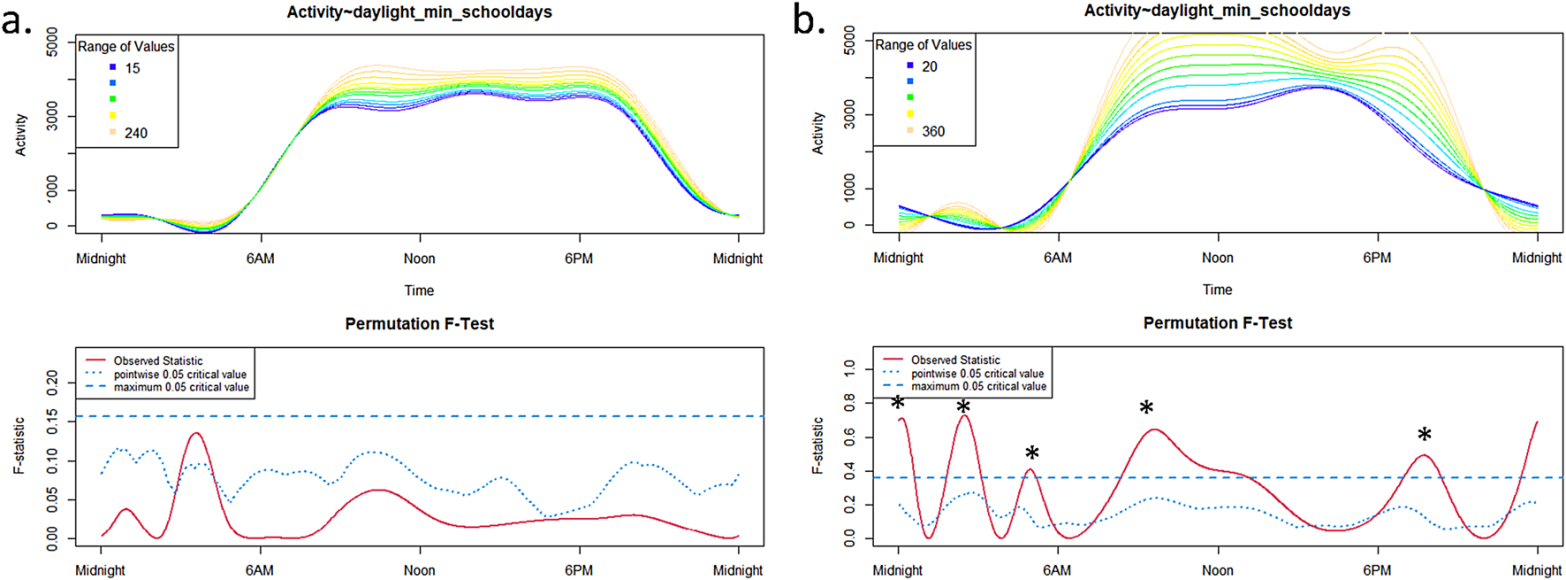
Influence of daylight exposure in the ADHD group (**a**) and in the TD group (**b**) on schooldays.

Differences between groups regarding age, IQ and sex were analyzed with IBM SPSS (version 27). To explore differences in 24h-actigraphy data between children with ADHD and TDs, the FLM method (Fourier expansion model (*n* = 9 Fourier basis permutation) fitted at a 24h periodicity^18^ using the package ‘Actigraphy’ (version 1.4.0) in R (version 4.0.4) was applied. To explore whether and when the groups’ activity profiles differed the set of 24h functional circadian activity patterns deriving from the Fourier expansion model were compared through non-parametric permutation F tests. ‘Group’ and ‘ADHD presentation’ were included as covariates. Analyses were calculated separately for the two groups (ADHD and controls) for each correlate and predictor of interest: ‘chronotype’, ‘light exposure’ and ‘toddler sleep problems’. FLM results are reported in Figs. 1, 2, 3, 4 and 5.

## Results

### Participant characteristics

ADHD children and TDs did not differ with regard to age, sex or chronotype. However, the ADHD group had higher ADHD symptom levels and a lower IQ (see Table 1).

**Table 1:** Demographic information.

	ADHD *n* = 35	TDs *n* = 39	Test statistic	*p*
Age (M ± SD)	9.94 ± 2.02	9.14 ± 1.70	F _1,72_ = 0.755	0.066
Sex (m/f)	27/8	26/13	X^2^ (1) = 0.996	0.318
IQ (M ± SD)	105.17 ± 13.90	115.44 ± 11.90	F _1,72_ = 1.143	0.001*
ADHD symptom score (M ± SD)	1.97 ± 0.39	0.19 ± 0.17	F _1,72_ = 22.242	< 0.001*
Chronotype (Md ± SD)	3.07 ± 0.90	2.71 ± 0.74	t(71) = -1.824	0.072

*p < 0.05.

*TD* typically developing peers, *m* masculine/boys, *f* feminine/girls, *SD* standard deviation, *M* mean, *Md* median.

In total, 77% of the children diagnosed with ADHD were medicated (see Table 2), most of them with an extended-release methylphenidate medication with duration of action usually lasting between 8 and 12 h.

**Table 2:** ADHD presentations and medication status.

	ADHD group			
	Combined presentation *n* = 19	Inattentive presentation *n* = 12	Hyperactive/impulsive presentation *n* = 4	Total *n* = 35
ADHD medication	17/19	8/12	2/4	27/35

### Group differences in 24h-activity profile between ADHD and controls

There was no significant between-group difference (*p* > 0.05) for the 24h-activity profile between ADHD and TDs, neither on schooldays (Fig. 1a) nor on free days (Fig. 1b).

The upper panels show the functional forms of the 24h-activity profile of ADHD and TD groups with ‘Activity’ (y-axis) and ‘Time’ (x-axis). The graphs below show the results of the permutation tests (F-tests). A significant between-group effect in the 24h-activity profile is indicated by a red solid line (the observed statistic) above the blue dashed line (the global test of significance with alpha set to 0.05).

We also conducted a subgroup analysis of only children with a combined or hyperactive/impulsive presentation in the ADHD group (Supplementary Table 1) as included in our recently published study^10^ focusing on sleep parameters (Supplement 2). Between-group comparison on the 24h-activity profile remained non-significant, despite to the significant SOL group differences found in the previous study using standard statistical analyses^10^ (Supplementary Fig. 1).

### Differences between ADHD presentations

The 24h-activity profiles of ADHD presentations differed significantly on free days (not on schooldays, Fig. 2a) at around 08:00 p.m. (Fig. 2b), with higher activity (more intensity) in the combined presentation compared to the inattentive presentation. However, since the subgroup of hyperactive/impulsive children is very small (*n* = 4), the result should be interpreted with caution.

The upper panels show the functional forms of the 24h-activity profile of ADHD and TD groups with ‘Activity’ (y-axis) and ‘Time’ (x-axis). The graphs below show the results of the permutation tests (F-tests). A significant between-group effect in the 24h-activity profile is indicated by a red solid line (the observed statistic) above the blue dashed line (the global test of significance with alpha set to 0.05).

### Correlates and predictors of the 24h-activity profile

#### Chronotype

For the ADHD group on schooldays (Fig. 3a), we found significant effects of the chronotype on 24h-activity profile during nighttime (~ 02:00 a.m.: early chronotypes show higher activity and ~ 04:00 a.m.: late chronoytpes show higher activity), in the morning ~ 07:00 a.m. (early chronotypes show higher activity), and in the evening ~ 11:00 p.m. (late chronoytpes show higher activity).

For the control group on schooldays (Fig. 3b), significant effects of the chronotype on 24h-activity profile were only revealed at ~ 10:00 a.m. (early chronotypes show higher activity) and ~ 11:00 p.m. (late chronoytpes show higher activity).

The upper panels show the functional forms of the 24h-activity profile of ADHD and TD groups with ‘Activity’ (y-axis) and ‘Time’ (x-axis). The graphs below show the results of the permutation tests (F-tests). A significant between-group effect in the 24h-activity profile is indicated by a red solid line (the observed statistic) above the blue dashed line (the global test of significance with alpha set to 0.05).

For the ADHD group (Fig. 4a), on free days significant effects of the chronotype on 24h-activity profile during nighttime (~ 02:00 a.m.: early chronotypes show higher activity and ~ 04:00 a.m.: late chronoytpes show higher activity), in the morning ~ 07:00 a.m. (early chronotypes show higher activity) and before midnight ~ 11:00 p.m. (late chronoytpes show higher activity) were observed. Additionally, in the evening ~ 07:00 p.m. (early chronotypes show higher activity) an influence of the chronotype on 24h-activity profile was apparent for the ADHD group.

The graph for the control group on free days (Fig. 4b) only showed a significant effect from ~ 08:00–11:00 a.m. (early chronotypes show higher activity).

The upper panels show the functional forms of the 24h-activity profile of ADHD and TD groups with ‘Activity’ (y-axis) and ‘Time’ (x-axis). The graphs below show the results of the permutation tests (F-tests). A significant between-group effect in the 24h-activity profile is indicated by a red solid line (the observed statistic) above the blue dashed line (the global test of significance with alpha set to 0.05).

##### Post-hoc analysis

In general, younger children showed higher activity in the mornings whereas older children moved more at the evening (ADHD: on schooldays; TD group: on free and schooldays, *p* > 0.05). Only in the ADHD group the significant effect that older children showed higher activity at the evening disappeared on free days. The influence of age on 24h-activity profile is displayed in detail in Supplementary Figs. 2 and 3 online.

Younger age corresponded to an earlier chronotype in the ADHD group (*r* = 0.66, *p* < 0.001) and in TDs (*r* = 0.40, *p* = 0.012).

#### Daylight exposure

A significant effect (*p* < 0.05) of daylight exposure on the 24h-activity profile was found only in the TD group on schooldays (Fig. 5b) around midnight, ~ 02:00 a.m. (more daylight exposure corresponds to higher activity), ~ 05:00 a.m. (less daylight corresponds to higher activitiy), ~ 09:00–10:00 a.m. (more daylight exposure corresponds to higher activity) and around 07:00 p.m (more daylight exposure corresponds to higher activity). There was no influence for ADHD children (Fig. 5a).

##### Post-hoc analysis

TD children spent significantly more time (1 h) outside on schooldays (TD: m = 2 h 46 min ± 85.556 min; ADHD: 1 h 46 min ± 51.679 min; t(72) = 3.561, *p* = 0.001) and were exposed to daylight for a significantly longer time (1 h 25 min) on free days (TD: m = 4 h 20 min ± 110.191 min; ADHD: 2 h 55 min ± 92.235 min; t(72) = 3.587, *p* = 0.001).

#### Sleep problems at toddler age and current sleep problems (CBCL)

**Fig. 6:**
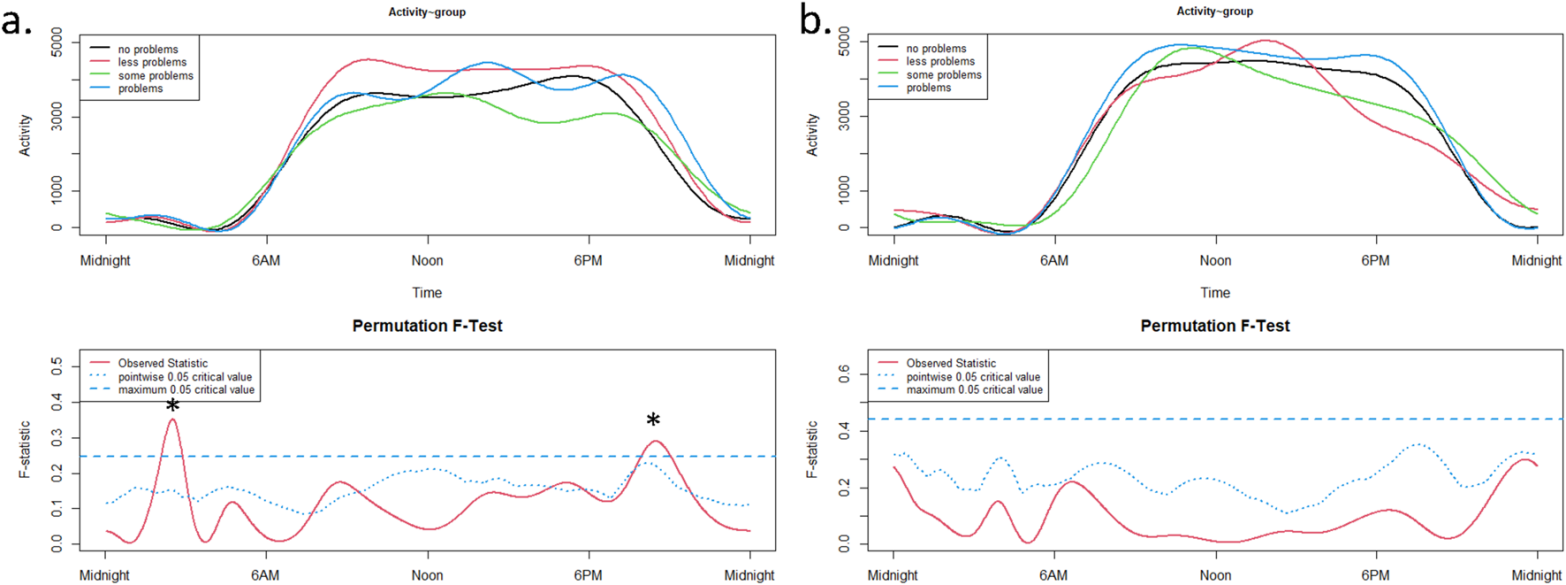
Influence of sleep problems at toddler age in the ADHD group (**a**) and in the TD group (**b**) on schooldays.

The association of sleep problems at toddler age on current 24h-activity profile was apparent only in the ADHD group on schooldays during a specific time window in the night (~ 01:00–03:00 a.m.: having sleep problems at toddler age corresponds to higher activity) and during bedtime (~ 09:00 p.m.: having sleep problems at toddler age corresponds to higher activity; Fig. 6a). In the TD group, there was no significant association of sleep problems at toddler age on 24h-activity profile (*p* > 0.05; Fig. 6b).

Current sleep problems did not affect the 24h-activity profile of the children, neither in the ADHD nor in the TD group (all *p* > 0.05).

The upper panels show the functional forms of the 24h-activity profile of ADHD and TD groups with ‘Activity’ (y-axis) and ‘Time’ (x-axis). The graphs below show the results of the permutation tests (F-tests). A significant between-group effect in the 24h-activity profile is indicated by a red solid line (the observed statistic) above the blue dashed line (the global test of significance with alpha set to 0.05).

##### Post-hoc analysis

We observed positive correlations between sleep problems at toddler age and current sleep problems in both groups (TD: *r* = 0.59, *p* < 0.001; ADHD: *r* = 0.58, *p* = 0.001), indicating that higher levels of sleep problems at toddler age are associated with more current sleep problems. The distributions of current sleep problems and sleep problems in toddler age can be found in Supplement 4 online (Supplementary Table 2). 37% of ADHD children showed problems at toddler age versus 24% of TD children (ꭓ^2^ (1) = 1.57, *p* = 0.211). In the CBCL, 34% of children with ADHD reported sleep problems, but only 12% of children without ADHD (ꭓ^2^ (1) = 4.53, *p* = 0.033).

## Discussion

This study found neither overall nor time-specific differences in 24h-motor activity profiles between children with and without ADHD. However, differences between ADHD presentations indicate that 24h-activity profiles might be useful for ADHD subtype characterization. Different correlational and predictive effects were found for each group indicating differential effects for ADHD patients versus TDs and probably distinct underlying mechanism.

The overall results showing no between-group effects partially contradict the findings of Tonetti et al.^16^, who found significantly higher mean motor activity across the whole day and during specific time periods (2:00–3:00 a.m.) at night in the ADHD group. However, our results are in line with the finding that FLM indicates no group difference around the time of sleep onset (despite the SOL differences in our previous study^10^. In contrast to our study, the sample of ADHD children in the Tonetti study was treatment-naïve. Due to the guideline-based, stepped-care design of our multicenter study from which participants were recruited, most of the ADHD children were medicated and had also previously received psychoeducation, both probably having an impact on motor activity. With regard to the medication effects, especially a diminished hyperactivity during daytime (lower levels of symptoms of hyperactivity) has been replicated several times (e.g.^43,44^). However, there is also an ongoing debate about a potentially (negative) influence of ADHD medication on sleep problems and/or circadian rhythm aberrations caused by medication (e.g., insomnia is listed as a common adverse reaction in the prescribing information for stimulant medications^45^). Consequently, the non-significant between-group results could also be due to medication effects in children with ADHD having decreased their inherent daytime hyperactivity. Still, more studies are needed to shed light on the mechanisms associated with ADHD medication related sleep disturbances/circadian rhythm deviations, since the results of previous studies are mixed^46–50^. Especially dopaminergic functioning, which is important in the etiology of ADHD as well as in sleep regulation^4^, is of special interest for a better understanding of ADHD symptoms and the implementation of an adequate psychopharmacological treatment^8,51^. Additionally, previous studies already revealed that psychoeducation is a highly effective ingredient in the treatment of ADHD children (e.g^52–54)^. Children with ADHD and their parents may already successfully use some strategies to calm down during the afternoons or in the evenings and therefore differ less from the control children in our study. Another possible reason might be that the children with ADHD included in the Tonetti study were not age and gender matched. This resulted in a control sample that was significantly older and had a higher proportion of girls, which may have driven group differences in their study, since older participants and girls would be expected to show less activity during day.

Comparing the different ADHD presentations, we found significantly higher activity for the combined presentation in the 24h-activity profile on free days at around 08:00 p.m. This effect occurring around bedtime might be related to sleep onset problems or probably directly reflect difficulties in falling asleep. This finding is partly in line with the results from Tonetti et al.^16^, who also reported higher activity in the combined (and hyperactive/impulsive) presentation compared to the inattentive presentation in a specific time window in the evening and during the night. However, on school days the difference between the ADHD presentations around sleep onset time diminishes, which may be related to a more consistent bedtime routine carried out by the parents during schooldays in contrast to free days in ADHD families^55^. Further, the non-significant differences between ADHD presentations during the day in general and specifically in the mornings could be explained by the fact that 90% of the patients in our group with combined presentation were medicated, which may have even led to less (hyper)activity compared to TDs. However, the sample sizes of the subgroups were small, especially for the hyperactive/impulsive presentation, and not balanced, so the results need to be interpreted with caution and require replication in larger samples.

The chronotype on free days turned out as one important correlate of the 24h-activity profile in the ADHD group. Other significant effects found for the TD group and on school days across groups might be related to age (see respective analyses with age as covariate in Supplement 3 online) and are therefore not specific for the chronotype as age and chronotype are highly correlated. There were no significant differences in the chronotype between children with and without ADHD, in contrast to the findings from some other previous studies in children with ADHD (e.g^56^) or adult ADHD patients (e.g^26)^. For the assessment of the chronotype, we used a subjective questionnaire (MCTQ) which does not assess the child’s sleep time preference but rather the actual bedtimes that are usually regulated by parents. However, there is an association between the chronotype and the 24h-activity profile in the ADHD group on free days, as expected with higher activity in the later chronotype around bedtime, again supporting the hypothesis of a stricter regulation of sleep/bedtime routines by the parents during the week compared to the weekend (e.g^55)^. In turn, the late chronotype of children with ADHD has a stronger impact during weekends (or vacations), as ADHD children can plan their day more in accordance with their chronotype, which is reflected in more movements in the evening.

Further, daylight influenced the 24h-activity profile on schooldays only in TD children whereas in the ADHD group we could not find this effect. The non-significant result could also be caused by a lower variance in the ADHD group compared to TD children. As shown in post-hoc analyses, children with ADHD spent significantly less time outside on schooldays as well as on free days compared to TD children. The causes for this difference might be diverse: first, many children with ADHD spend more time on their homework during the afternoon due to their attentional deficits and especially when their ADHD medication loses its effect. That in turn leads to less free time and probably less time to be spend outside. Second, children with ADHD are more prone to excessive media consumption, as for example computer games, and so potentially spend more time indoors than playing with friends outside (e.g^57^). Third, a non-negligible proportion of parents of ADHD children show ADHD-like symptoms themselves and may have difficulties giving their children the appropriate structure to plan afternoons, including time spend outside^58^. Sleep hygiene aspects in general (e.g. an adequate structure of the day with less or no screen time in the evenings, recurring bedtime routines or positive parenting domains like consistency or warmth) represent modifiable factors in the management of circadian rhythm shifts in families with ADHD^55^. Our results show a significant difference in the time spent outside and, consequently, the amount of contact with natural daylight between children with and without ADHD. This finding aligns with the idea of using light therapy for patients with ADHD, since a few earlier studies already indicate positive effects of daylight exposure on symptoms of ADHD (e.g^59)^. But still, studies in large and well characterized samples that provide clear evidence for the usability of (bright) light exposure for improving symptoms of ADHD are scarce^8,60^, even though there are some promising results.

The importance of early regulation problems as potential early markers for the emergence of later ADHD symptoms is supported by the current findings that sleep problems in toddler age are related to the 24h-activity profile, including sleep phases, in later childhood. A significant effect evolved during bedtime and during a specific time window at night (01:00–03:00 a.m.). In a previous study, we showed that sleep problems at toddler age are predictive for specific sleep problems in later childhood-age for children with and without ADHD, especially with regard to later sleep onset and general movements during sleep^10^. The results are further in line with previous studies that analyzed long-term effects of infant sleep problems on the development of ADHD at the age of 5.5 years^33^. The current study extends these findings by focusing not only on sleep phases but on the 24h-activity profile detecting the specific time window when higher motor activity occurs. However, the predictive value of sleep problems in toddler age needs to be investigated in future studies with longitudinal designs for more solid conclusions.

The results of this study need to be interpreted in the context of the following limitations: (1) the sample sizes of our groups are relatively small, especially with regard to the subgroup analyses of the different ADHD presentations. (2) The lack of an unmedicated group of children with ADHD is a further limitation owed to the stepped-care treatment design of our multicenter study from which patients were recruited. (3) Chronotype and daylight exposure are subjectively reported measures depending also on parent behavior and family routines and have not been assessed objectively. Nevertheless, the MCTQ questionnaire is an established and valid questionnaire representing a good correlate of dim light melatonin onset (DLMO), with the advantage of being easy to administer^24^. (4) For the assessment of toddler sleep problems and current sleep problems, we only used a single item of two different self-report questionnaires which carries the risk of memory distortion, especially for the toddler sleep problems. (5) The FLM method is just one model to address our research questions and limited to simple comparisons, not addressing interaction effects. With larger samples, more complex analyses might enable even deeper insights.

Even though we could not find general differences with regards to the 24h-activity profile between children with and without ADHD, many patients and their parents report sleep onset aberrations in line with previous studies and meta-analyses^61,62^. Currently, in the clinical routine of diagnosing ADHD, only little attention is paid to the assessment of sleep (problems) or an objective assessment of overt motor activity. However, the analysis of the real-life whole-day activity data could provide more comprehensive information about the system of the circadian rhythm (also known as the ‘biological clock’ (e.g^63)^ which influences multiple physiological processes like for example body temperature, heart rate or hormone secretion and consequently sleep behavior. Actigraphs could be used as an add-on diagnostic tool with the benefit of a cost-effective strategy to identify daily critical time periods where intervention recommendations and strategies are needed to also improve ADHD symptoms^64^.

Future FLM-based actigraphy studies should include larger samples of ADHD patients balanced for ADHD presentations and medication status and preferably longitudinal designs to detect predictors for early preventive action of sleep problems.

In our FLM-analysis, children with ADHD did not differ from TD peers in the 24h-activity profile. The lack of between-groups differences regarding the 24h-activity profile contrasts partially with differences in SOL and other sleep-related measures in a subsample of these participants. One major limitation of this study is that the majority of participants with ADHD were receiving pharmacological treatment, thereby limiting the generalizability of the results. However, it needs to be taken into account that SOL reflects a combination of objective actigraphy and subjective reports and this approach includes a different methodological procedure, which should be understood as complementary^10^. The significant FLM-differences between ADHD presentations on free days around bedtime revealed higher activity in the combined compared to the inattentive presentation. Although the small sample sizes in the subgroups limit the generalizability of the findings, they indicate that a potential diagnostic benefit may exist, warranting further research and replication. The analysis of early predictors and correlates of the 24h-activity profile provides insights into the complex mechanisms related to motor activity and sleep in patients with ADHD and might stimulate future studies, not only for a more holistic understanding of lifestyle habits but also for the development of more targeted interventions within a personalized medicine framework. In sum, the results underline the complementary value of a 24h-activity assessment and analysis by showing an objective 24h-profile of (hyper)activity, at least for ADHD subtyping.

## Supplementary Information

Below is the link to the electronic supplementary material.

Supplementary Material 1

## Data Availability

The datasets generated and analysed during the current study are not publicly available due to currently applicable privacy policy and in accordance with the informed consent statement of the participants, but are available from the corresponding author on reasonable request.
