# Supplementary material for: Insights from 24-hour actigraphy using functional linear modeling in children with and without ADHD
Source: Sci Rep. 2025 Oct 23;15:37161. doi: 10.1038/s41598-025-24040-5 (PMC12550039; doi:10.1038/s41598-025-24040-5)
Supplement: Supplementary file 1 — Supplementary Material 1 [file 41598_2025_24040_MOESM1_ESM.pdf]

**Supplement**

**Supplement 1: Methods details**

*Details of exclusion criteria:* Exclusion criteria were IQ < 80, insufficient German language and reading skills of parents/legal representatives with respect to relevant assessments (e.g., filling out questionnaires) and therapeutic interventions. Additionally, children with a diagnosis of pervasive developmental disorder, schizophrenia, bipolar disorder, severe depressive episodes, epilepsy, heart disease, and current or planned intensive behavioral therapy for ADHD or oppositional behavior (ODD) on a weekly basis were excluded. Further exclusion criteria were a known non-response to standard ADHD medication (methylphenidate, (lis)dexamphetamine, and atomoxetine), psychotropic medication (other than for ADHD), or neuroleptic medication (other than for the treatment of disturbance of impulse control) in line with the ESCAschool study protocol, including also pharmacotherapeutic interventions.

*Diagnose-Checklist ADHS (DCL–ADHS–clinical interview):* This checklist measures symptoms of ADHD according to the diagnostic criteria of the DSM-5 and the International Classification of Diseases 10th edition (ICD-10) and is recommended in the German guidelines for the assessment of ADHD symptoms and treatment evaluation. Three ADHD presentations can be distinguished by using this checklist: combined presentation, predominantly inattentive presentation, predominantly hyperactive-impulsive presentation.

**Supplement 2: FLM analysis with data from Ziegler et al. (2021)**

a) b)

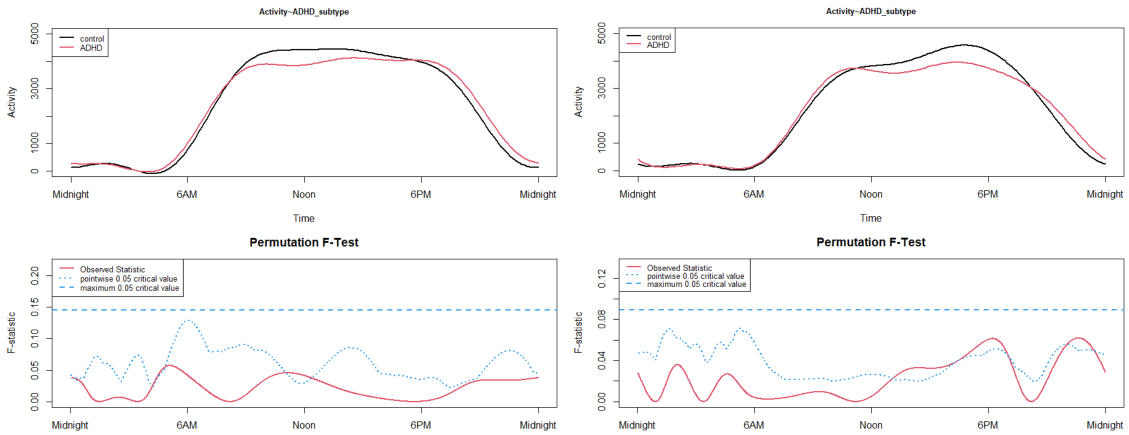


**Supplementary Figure 1.** 24h-activity profile of ADHD and control group from Ziegler et al. (2021) on schooldays (a) and on free days (b) indicating no significant effects ($p > 0.05$)
The upper panels show the functional forms of the 24h-activity profile of ADHD and TD groups with 'Activity' (y-axis) and 'Time' (x-axis). The graphs below show the results of the permutation tests (F-tests). A significant between-group effect in the 24h-activity profile is indicated by a red solid line (the observed statistic) above the blue dashed line (the global test of significance with alpha set to 0.05).

**Supplementary Table 1**. Demographic Information from the sample from Ziegler et al. (2021)

| | ADHD *n* = 24 | TDs *n* = 33 | Test Statistic | *p* |
|---|---|---|---|---|
| Age (M ± SD) | 9.66 ± 1.95 | 9.18 ± 1.64 | $F_{1,55} = 0.182$ | 0.32 |
| Sex (m/f) | 20/4 | 22/11 | $X^2$ (1) = 12.789 | <0.001[1] |
| IQ (M ± SD) | 106 ± 14.7 | 118 ± 10.8 | $F_{1,55} = 3.389$ | <0.001[1] |
| ADHD symptom score (M ± SD) | 2.16 ± 0.35 | 0.20 ± 0.18 | $F_{1,55} = 2.670$ | <0.001[1] |
| Combined subtype | 20/24 | / | | |
| Hyp/Imp. subtype | 4/24 | / | | |
| Comorbid ODD | 11/24 | / | | |

[1] The groups differed significantly regarding sex and IQ as well as regarding ADHD-specific markers

**Supplement 3. 24h-activity profile and age (in months)**

*Schooldays:* The graphs showed higher activity in the ADHD group in younger children in the mornings at around 08:00 a.m. and in the evenings around 06:00 p.m.; at night it reverses when older children showed higher activity (around 02:00 a.m. and 04:00 a.m.). In the TD group younger children moved more from around 09:00 a.m. till 04:00 p.m. and older children showed higher activity in the evenings till midnight.

a) b)

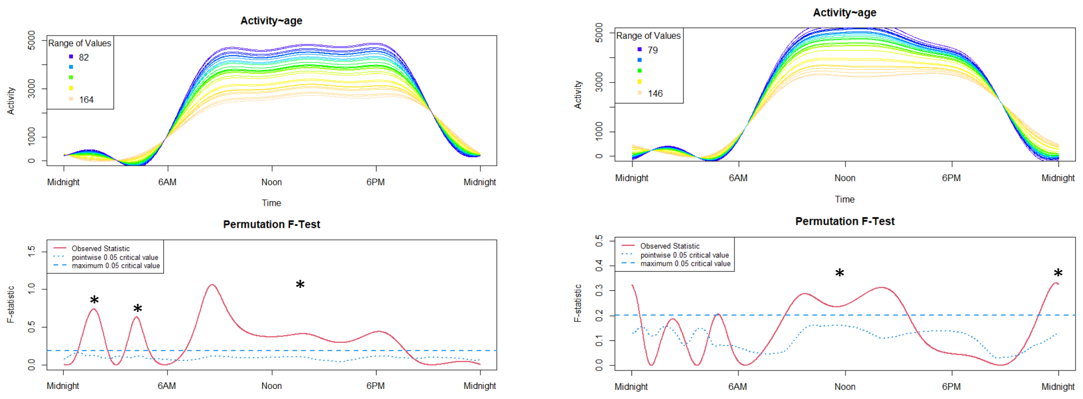


**Supplementary Figure 2, schooldays.** Influence of age on 24h-activity profile in the ADHD group (a) and in the TD group (b)

The upper panels show the functional forms of the 24h-activity profile of ADHD and TD groups with 'Activity' (y-axis) and 'Time' (x-axis). The graphs below show the results of the permutation tests (F-tests). A significant between-group effect in the 24h-activity profile is indicated by a red solid line (the observed statistic) above the blue dashed line (the global test of significance with alpha set to 0.05).

*Free days:* In the ADHD and in the TD group younger children showed higher activity in the mornings. Around 06:00 p.m. the difference between younger and older children was significant only in the ADHD group whereas the TD children showed again higher activity in the evenings till midnight and specific time periods in the nights (Figure 3).

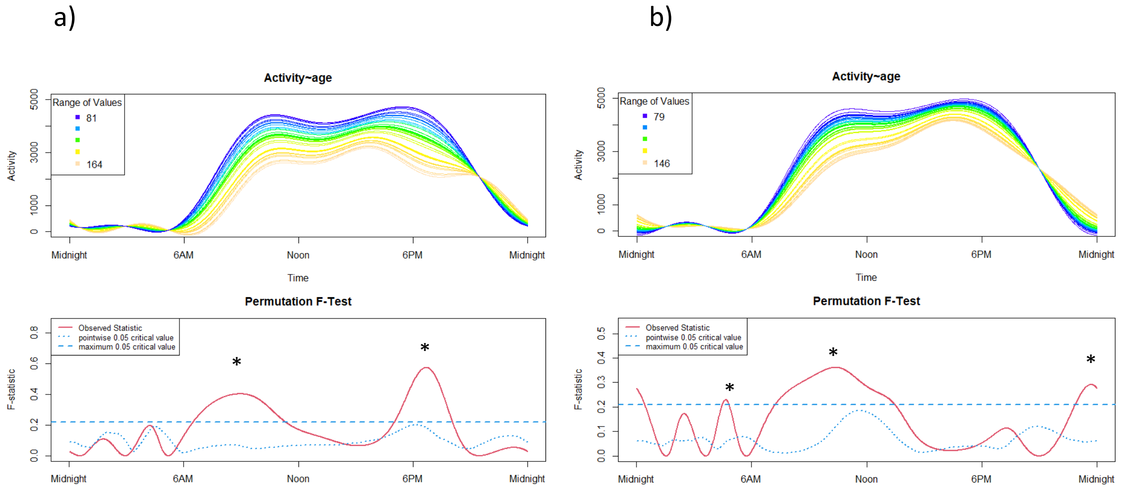


**Supplementary Figure 3, free days.** Influence of age on 24h-activity profile in the ADHD group (a) and in the TD group (b)

The upper panels show the functional forms of the 24h-activity profile of ADHD and TD groups with 'Activity' (y-axis) and 'Time' (x-axis). The graphs below show the results of the permutation tests (F-tests). A significant between-group effect in the 24h-activity profile is indicated by a red solid line (the observed statistic) above the blue dashed line (the global test of significance with alpha set to 0.05).

**Supplement 4. Sleep problems at toddler age (DOTS-R) and in childhood-age (CBCL).**

**Supplementary Table 2.**

| **Toddler sleep problems (DOTS-R)** | **ADHD N=35** | **TDs N=38** |
|---|---|---|
| **no problems** | 17 (49%) | 25 (66%) |
| **less problems** | 5 (14%) | 4 (11%) |
| **some problems** | 6 (17%) | 6 (16%) |
| **problems** | 7 (20%) | 3 (8%) |

| **Current sleep problems (CBCL)** | **ADHD N=32** | **TDs N=33** |
|---|---|---|
| **no symptoms** | 21 (66%) | 29 (88%) |
| **sometimes** | 2 (6%) | 3 (9%) |
| **frequently** | 9 (28%) | 1 (3%) |
